# Supplementary figures and images for: Discordant rearrangement of primary and anamnestic CD8+ T cell responses to influenza A viral epitopes upon exposure to bacterial superantigens: Implications for prophylactic vaccination, heterosubtypic immunity and superinfections
Source: PLoS Pathog. 2020 May 20;16(5):e1008393. doi: 10.1371/journal.ppat.1008393 (PMC7239382; doi:10.1371/journal.ppat.1008393)

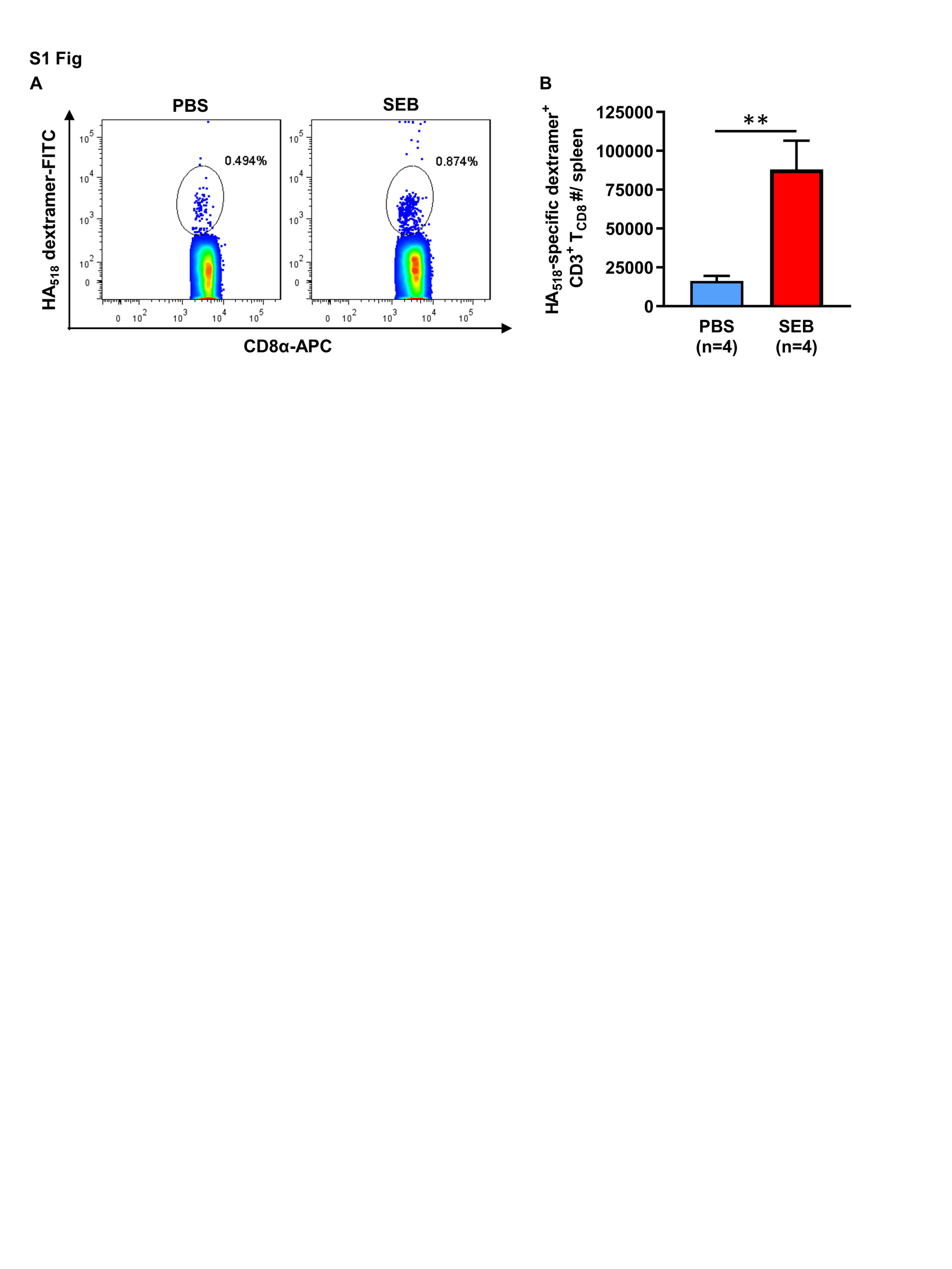

Supplement: S1 Fig — BALB/c mice were injected i.p. with PBS or with 50 μg of SEB three days before they were immunized i.p. with the PR8 strain of IAV. Seven days later, HA518-specfic TCD8 were identified through surface staining with an anti-CD8α mAb and MHC I dextramers. Representative dot plots after live gating on CD3+ events are demonstrated in panel A. In addition, the absolute numbers of splenic HA518-specific cells were calculated (B). Data are shown as mean ± SEM for 4 mice per group. ** denotes a statistically significant difference with p<0.01 using an unpaired Student’s t-test. (TIF) [file ppat.1008393.s001.tif]

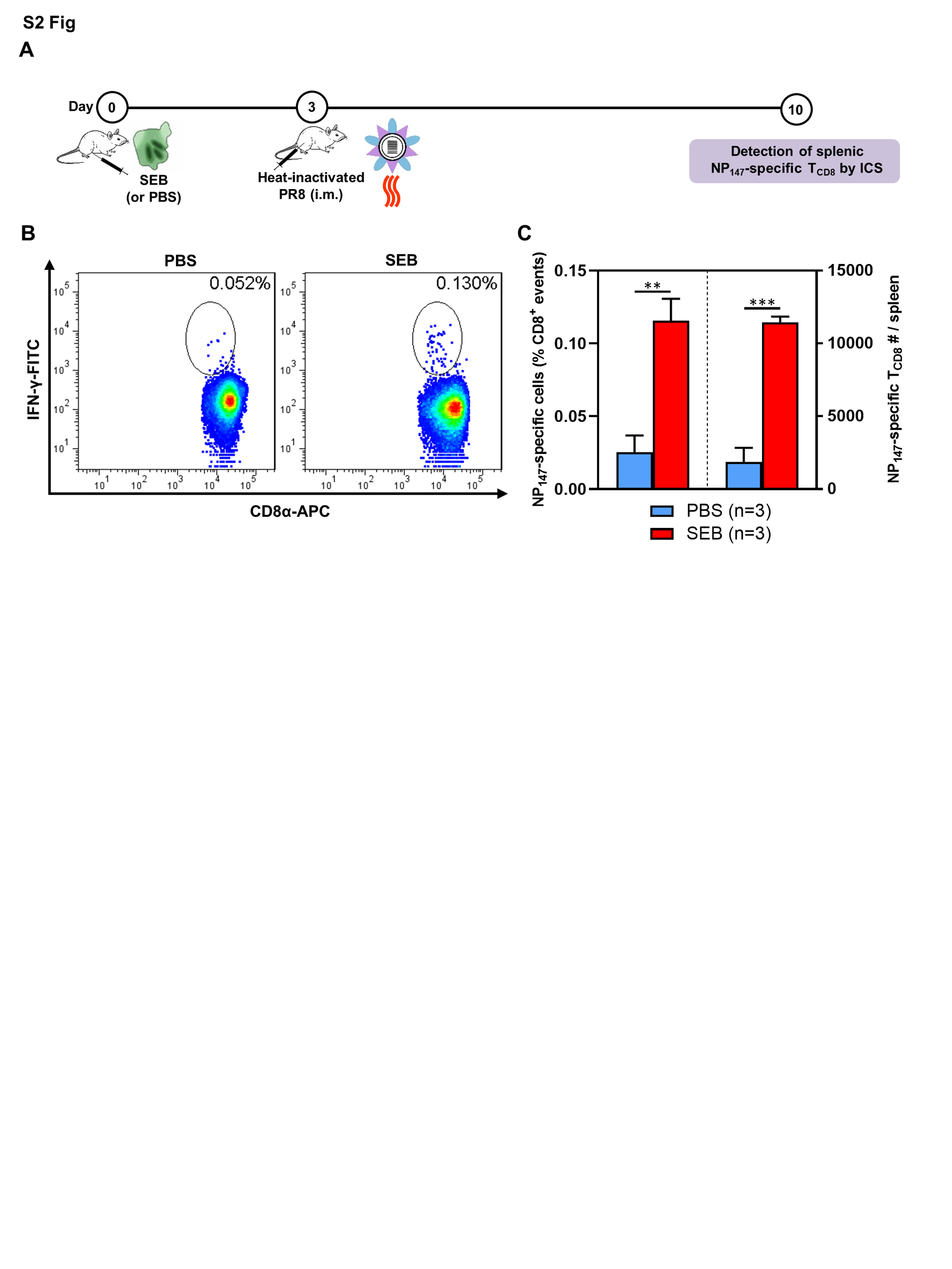

Supplement: S2 Fig — (A) Mice were injected i.p. with PBS or with 50 μg SEB followed, three days later, by i.m. vaccination with heat-inactivated PR8. Seven days after vaccination, the frequency (B,C) and the absolute number (C) of NP147-specific TCD8 was determined in each spleen by ICS for IFN-γ. Representative dot plots (B) and summary data (C) are depicted. Error bars represent SEM, and ** and *** denote statistically significant differences with p<0.01 and p<0.001, respectively, using an unpaired Student’s t-test. (TIF) [file ppat.1008393.s002.tif]

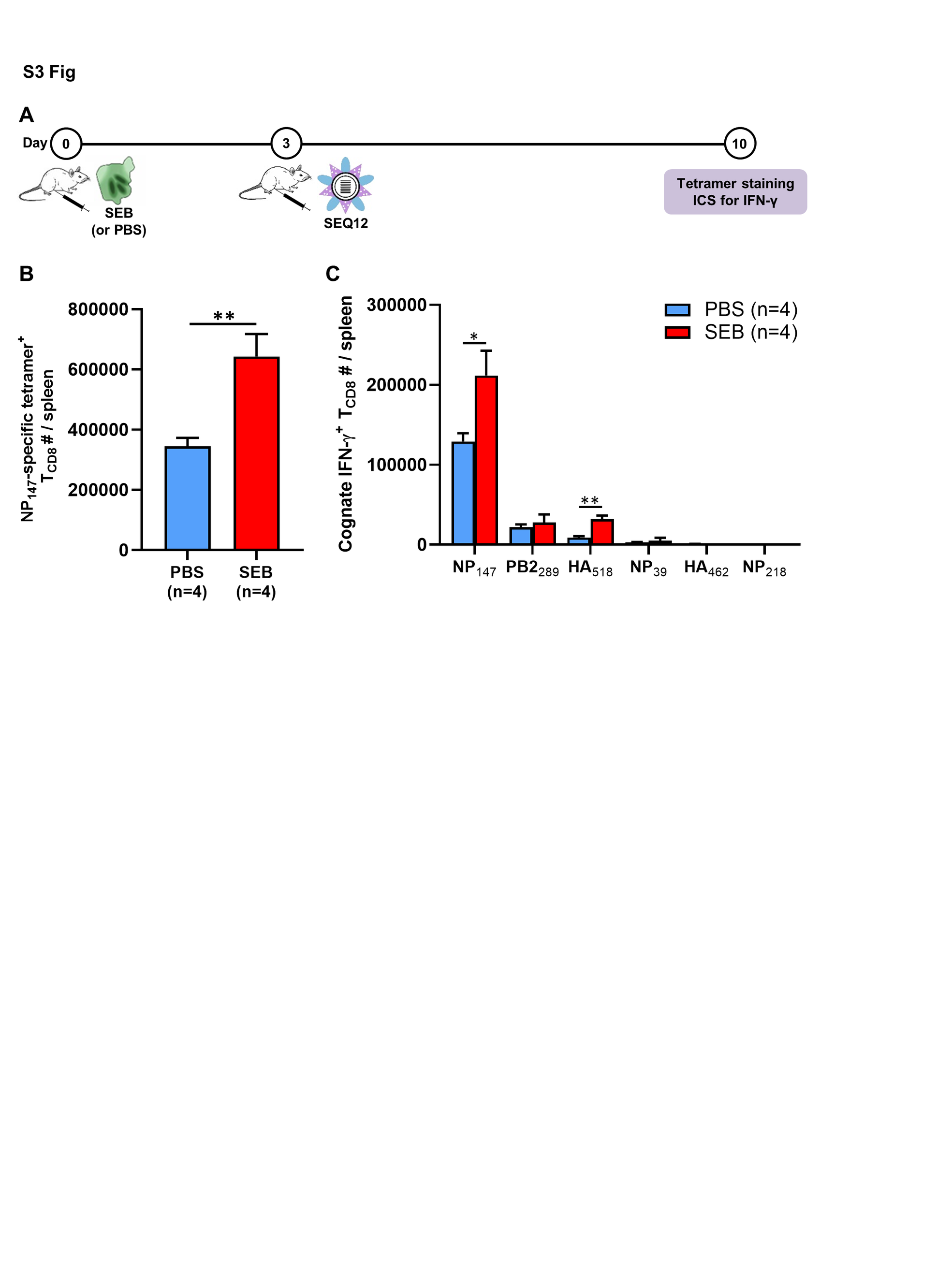

Supplement: S3 Fig — (A) BALB/c mice (n = 4/group) were injected i.p. with PBS or with 50 μg SEB three days before they were immunized i.p. with SEQ12. At the peak of the primary response (i.e., on day 7 post-immunization), TCD8 recognizing the indicated epitopes were enumerated by MHC I tetramer staining (B) and by ICS for IFN-γ (C). Error bars represent SEM. * and ** denote statistically significant differences with p<0.05 and p<0.01, respectively, which were determined using an unpaired Student’s t-test (TIF) [file ppat.1008393.s003.tif]

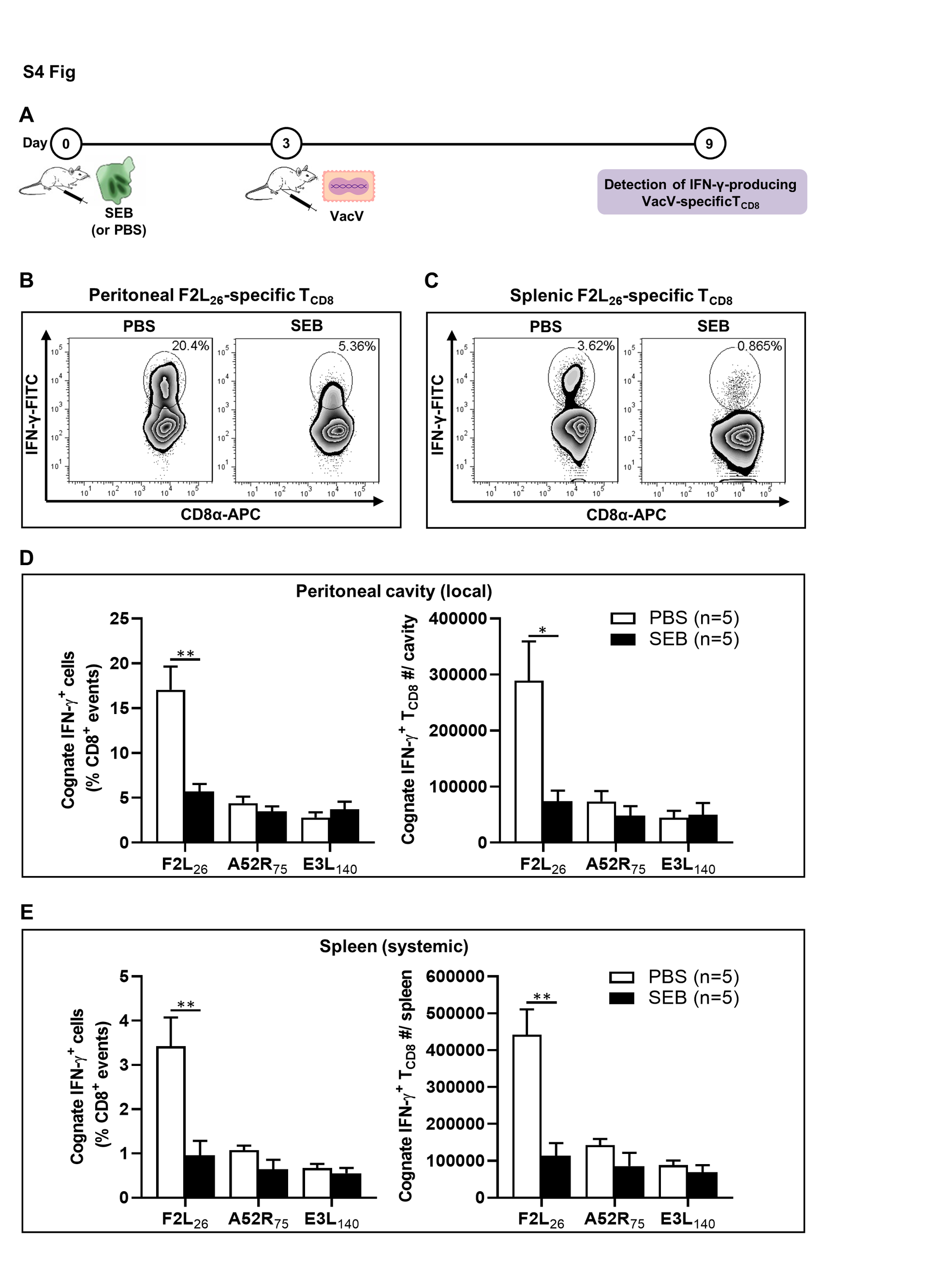

Supplement: S4 Fig — (A) Mice were injected i.p. with PBS or with 50 μg SEB three days before they received VacV i.p. On day 6 post-immunization, a time point at which VacV-specific TCD8 responses reach their peak, peritoneal (B,D) and splenic (C,E) responses to indicated peptides were quantified by ICS for IFN-γ. Representative zebra plots in B and C illustrate the frequencies of peritoneal (local) and splenic (systemic) TCD8 responses to F2L26, respectively. Summary data from PBS- and SEB-treated mice (n = 5/group pooled from two independent experiments) also depict subdominant responses to A52R75 and E3L140 (D,E). Error bars represent SEM, and * and ** denote significant differences with p<0.05 and p<0.01, respectively, using an unpaired Student’s t-test. (TIF) [file ppat.1008393.s004.tif]

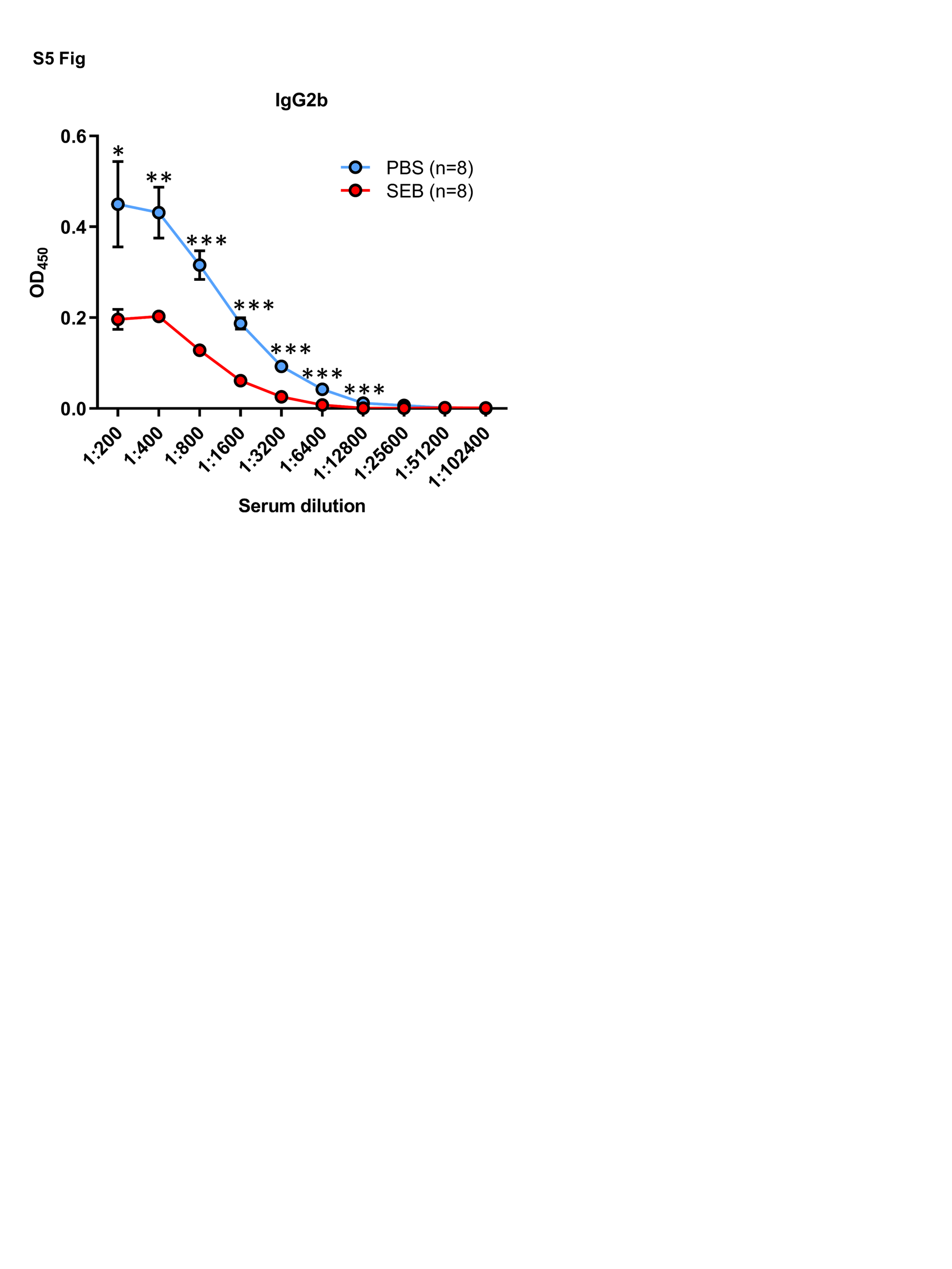

Supplement: S5 Fig — BALB/c mice were infected i.n. with 0.3 MLD50 of PR8 three days after they received an i.p. injection of PBS or SEB (50 μg). Three weeks after PR8 infection, mice were terminally bled, and the presence of PR8-specific IgG2b was evaluated in serially diluted serum samples as described in Materials and Methods. Error bars represent SEM. *, ** and *** denote statistically significant differences with p<0.05, p<0.01 and p<0.001, respectively. (TIF) [file ppat.1008393.s005.tif]
