# Supplementary material for: Discordant rearrangement of primary and anamnestic CD8+ T cell responses to influenza A viral epitopes upon exposure to bacterial superantigens: Implications for prophylactic vaccination, heterosubtypic immunity and superinfections
Source: PLoS Pathog. 2020 May 20;16(5):e1008393. doi: 10.1371/journal.ppat.1008393 (PMC7239382; doi:10.1371/journal.ppat.1008393)
Supplement: Attachment — Submitted filename: Meilleur et al.Responses to Reviewers.docx [file ppat.1008393.s006.docx]

**Point-by-Point Responses to Review Comments**

Dear Drs. Miller, García-Sastre, Haldar and McFadden,

**RE: Our revised manuscript entitled “Discordant rearrangement of primary and anamnestic CD8^+^ T cell responses to influenza A viral epitopes upon exposure to bacterial superantigens: implications for prophylactic vaccination, heterosubtypic immunity and superinfections”**

Many thanks for granting us the opportunity to submit a revised version of the above manuscript and also for extending our deadline to enable completion of the requested experiments. We were pleased to learn that “*the reviewers appreciated the attention to an important problem*” and that “*the reviewers largely appreciated the robustness of the presented data*”. We would like to express our sincere gratitude towards *PLOS Pathogens* editors and the reviewers of our work whose insightful comments and critique enabled us to improve our manuscript. As instructed in your decision letter, we have now provided our point-by-point responses below and described how we have revised our manuscript accordingly. These revisions involve inclusion of substantial new experimental data (forming 5 new figures and additional results detailed in the text), incorporation of 16 new references, and modification of the text to address reviewers’ comments. We hope these revisions will be satisfactory.

Sincerely,

S.M. Mansour Haeryfar

**Comments from Editor(s):**

“*Inactivated influenza should be administered i.m. to mimic inactivated influenza virus vaccination (and/or an attenuated influenza virus could be administered i.n. to mimic FluMist. Secondly, in the context of both infection and vaccination, SEB should be administered into the lungs at moderate levels, or better still, an SEB-expressing pathogen could [be] used to confirm the reported phenotypes*.”

Following all your instructions, we have now added substantial new data to our manuscript. More specifically, we have performed experiments in which heat-inactivated PR8 was administered intramuscularly as advised. We have tested the effect of intranasally instilled SEB in our PR8 infection model. We also obtained and utilized FluMist which, fortunately for us, generates a cross-reactive NP_147_-specific T cell response in BALB/c mice [Slütter B et al: *J Immunol* 2013, 190(8): 23854-8]. Finally, we used SEB-sufficient *Staphylococcus aureus* (strain COL) and an SEB deletion mutant of this strain (COL Δ*seb*) in additional new experiments. For details, please refer to our point-by-point responses below and see the highlighted parts of our revised manuscript.

**Comments from Reviewers:**

**Part I – Summary**

**Reviewer 1:**

“*Mice who are naïve to influenza, and therefore representing only very young children, are exposed to SEB by the i.p. route, and the influenza virus by the i.p. route. This does not model any situation that occurs in humans.*"

We have assessed the impact of exposure to bacterial superantigens (SAgs) on influenza A virus (IAV)-specific CD8^+^ T cell (T_CD8_) responses comprehensively, in naïve and also in previously immunized mice, and from several view angles. Anti-IAV T_CD8_ responses in previously naïve mice are commonly studied for many reasons, including but not limited to the examination of immunodominance hierarchies. As very correctly pointed out by the reviewer, pediatric populations may not have immunological memory to many IAV strains. In addition, when a completely new strain strikes, during seasonal or pandemic flu, there can be many individuals, vaccinated or unvaccinated, who lack immunological memory in order to quickly combat the new bug. Please note that our initial manuscript also contained data arising from experiments in which IAV-specific responses to a recall challenge (old Fig. 5D-F and Fig. 6, which are now Fig. 4D-F and Fig. 5, respectively) or to intranasally (i.n.) administered IAV (old Fig. 4 and Fig. S3, which are now Fig. 7 and Fig. S5, respectively) were quantitated. Even though intraperitoneal (i.p.) injection of IAVs has been routinely employed as a model of flu immunization for multiple reasons that are elaborated on in our revised manuscript (please read lines 351-362), we fully agree with the reviewer that using other injection routes and infection models are optimal. Therefore, we have now incorporated substantial new results from experiments in which heat-inactivated PR8 was administered intramuscularly (i.m.) (new Fig. S2), FluMist was instilled i.n. (lines 421-429), SEB and PR8 were co-administered i.n. (new Fig. 8), or infection with SEB-sufficient and -deficient *Staphylococcus aureus* was established via the i.n. route (new Fig. 9). Please also refer to the highlighted text in the revised manuscript.

“*The paper states that ‘IAVs constantly mutate to escape TCD8-medaited immunosurveillance’ without citing a reference. Where is the evidence that this is true? Antigenic drift definitely occurs in the surface glycoproteins but internal antigens are highly conserved.*”

We thank the reviewer for bringing up a very important subject. Although the internal components of IAVs, such as nucleoprotein (NP), are highly conserved, this does not necessarily mean that they never mutate. In fact, mutations in HLA-B27- and HLA-B35-restricted NP-derived peptides to facilitate escape from cytotoxic T lymphocyte (CTL) detection have been reported [Voeten JT, et al: *J Virol* 2000, 74(15): 6800-7; Boon AC, et al: *J Virol* 2002, 76(5): 2567-72]. Please also note that other than internal proteins, surface glycoproteins, namely hemagglutinin (HA) and neuraminidase (NA) that are often affected by antigenic drift, can also be recognized and responded to by T_CD8_. In our investigation, a T_CD8_ response to HA_518_ was detectable as expected, which could be augmented in SEB-treated mice. This was in addition to the immunodominant NP_147_-specific response. We have now briefly discussed the above points in our revised manuscript and cited two references as advised by the reviewer. As for a possible escape from T_CD8_-mediated immune surveillance, acute IAV infection is often, but not always, quickly cleared in immunocompetent hosts, which may leave little room for escape mutants to be generated. In a mouse model in which IAV persistence was observed after the acute infection was controlled, NP mutants that could no longer be detected by cognate CTLs emerged [Price GE, et al: *J Exp Med* 2000, 191(11): 1853-67]. Nevertheless, we agree with the reviewer that using the word “constantly” in the above statement may not always be applicable. Therefore, we have removed the sentence in question. We believe that the above changes helped us improve our manuscript, for which we are grateful to the reviewer.

“*There is very little evidence for ‘immunodominance’ of influenza virus epitopes in humans.*”

The polymorphic nature of HLA class I molecules, among other limitations, has historically resulted in less frequent studies on T_CD8_ immunodominance in humans. However, the significance of this phenomenon has been long suspected and is becoming increasingly clearer. Although many studies on IAV-specific T_CD8_ responses have been conducted in inbred mice, we think there is ample evidence indicating or strongly suggesting that immunodominance is relevant to human anti-IAV responses [Wu C, et al: *PNAS* 2011, 108(22): 9178-83; Grant E, et al: *Immunol Cell Biol* 2013, 91(2): 184-94; Grant EJ, et al: *Nature Commun* 2018, 9(1): 5427]. These references are now cited in our manuscript. There have been numerous more studies, for instance those led by Alessandro Sette, on the subject.

“*The researchers go on to assess SEB administration before immunization with 'rVVs'. Presumably recombinant Vaccinia viruses, which need a much better description in the methods section - what strain, and how are the minimal epitopes targeted to the ER (and also, why?) Why use epitopes only instead of whole antigens? Again, humans will never be immunized with rVVs expressing influenza epitopes. Humans will never be immunized by rVVs expressing whole influenza antigens either, and especially not by the i.p. route.*”

In our initial submission, we included the results we had obtained using recombinant vaccinia viruses (rVVs) encoding IAV epitopes as endoplasmic reticulum (ER)-directed minigenes. The primary purpose of these experiments was to evaluate the contribution of direct priming in generation of anti-IAV T_CD8_ responses. This was inspired by previous findings that such rVVs promote direct priming as opposed to cross-priming [Norbury CC, et al: *Science* 2004, 304(5675): 1318-21]. As advised by other reviewers, we have now removed this part from our current manuscript, and will use the data for a future submission. Instead, we have provided new data using wild-type vaccinia virus (VacV) (Western Reserve strain) for the purpose of exploring whether or not the impact of SEB on antiviral T_CD8_ responses is universal (please refer to new Fig. S4 and highlighted text). In addition, VacV-specific T_CD8_ clones fall into a well-established immunodominance hierarchy, which enabled us to address this phenomenon following immunization with a completely different virus. The natural route of VacV is unknown, and we and others have administered this virus via different routes, including i.p., in previous preclinical investigations [Tscharke DC, et al: *J Exp Med* 2005, 201(1): 95-104; Haeryfar SM, et al: *J Immunol* 2005, 174(6): 3344-51; Haeryfar SM, et al: *J Immunol* 2008, 181(1): 649-59; Maleki Vareki S, et al: *Am J Transplant* 2012, 12(1): 233-9; Memarnejadian A, et al: *J Immunol* 2017, 199(9): 3348-59]. It is also noteworthy that although smallpox is officially eradicated, prophylactic vaccination by VacV is still offered to at-risk military personnel and biomedical researchers working with poxviruses. Finally, poxviral and VacV-based vectors have been employed in vaccine formulations to protect against other infectious diseases, including influenza [Altenburg AF, et al: *Viruses* 2014, 6(7): 2735-61; de Vries RD, et al: *J Infect Dis* 2018, 218(4): 614-23; de Vries RD, et al: *Hum Vaccin Immunother* 2016, 12(11): 2881-2901]. Please note that our VacV results constitutes only a very small component of our manuscript. However, we believe they add an interesting new dimension to our work, which can be further pursued in future studies by us or by others.

**Reviewer 2:**

“*This is an interesting study that ….*”

“*In well designed and well executed experiments ….*”

“*Overall the issue is of some importance given the well-known but poorly understood interactions between IAV and bacterial infections in humans, that have also been explored to some extent in mouse models.*”

We thank reviewer 2 for his/her positive feedback.

“*This model using purified super antigens and IP IAV infection is rather oversimplified. When they infect intranasally the findings become quite complex. Overall it is not clear how relevant these findings will be in humans and that could be discussed and reviewed in more detail in the Discussion.*”

We appreciate the reviewer’s valid points. In addition to using purified SAgs, which would mimic clinical scenarios in which the toxins are present in the absence of bacteria (*e.g.*, during toxic shock), we have now added a new model in which SEB-sufficient and -deficient *Staphylococcus aureus* strains (COL and COL Δ*seb*) were used intranasally (i.n.) to mimic bacterial infections in human (please see new Fig. 9). As for injection/immunization routes, we have now added the results of new experiments in which SEB and PR8 were co-administered i.n. (please see new Fig. 8). To further boost our results, we used an additional model in which heat-inactivated PR8 was injected intramuscularly (please see new Fig. S2). Finally, we obtained and utilized FluMist, which is known to generate a cross-reactive NP_147_-specific response in BALB/c mice [Slütter B et al: *J Immunol* 2013, 190(8): 23854-8] (please refer to lines 421-429). Thanks of course to the reviewer’s insightful comments, these additional experiments have enriched our manuscript. Please also refer to the highlighted text in the Discussion and throughout the revised manuscript for additional details.

**Reviewer 3:**

“*Overall, the authors convincingly demonstrate that SEB administration prior to influenza virus administration increases the number of CD8 T cells with Vβ[8.]3 and Vβ8[.1/2] TCR.*”

We thank the reviewer for his/her encouraging remarks.

“*A major weakness in the study is the relevance of the model system used here - ip injection of infectious influenza virus is very different from intramuscular injection of inactivated vaccine or natural infection of the respiratory tract. Thus, the significance of these results in the context of co-infection or the impact of SEB on influenza virus specific CD8 T cell responses is not thoroughly addressed.*”

Point taken! We have now added new results arising from experiments in which inactivated and live attenuated IAVs were administered intramuscularly (i.m.) and intranasally (i.n.), respectively. We are pleased to report that as hypothesized, exposure to SEB enhanced the NP_147_-specific T_CD8_ response elicited by the i.m. injection of heat-inactivated PR8, which more closely simulates seasonal flu vaccination (please see new Fig. S2). Similarly, i.n. instillation of FluMist resulted in a greater pulmonary response to NP_147_ (please refer to lines 421-429). Of note, once administered to BALB/c mice, the A/California/7/2009 component of this human vaccine yields a PR8-cross-reactive NP_147_-specific response [Slütter B et al: *J Immunol* 2013, 190(8): 23854-8]. Finally, we utilized an infection model in which mice were inoculated i.n. with SEB-sufficient or -deficient *Staphylococcus aureus* strains followed by PR8 infection and NP_147_-specific T_CD8_ enumeration (please see new Fig. 9). We have updated our manuscript text accordingly.

**Reviewer 4:**

“*The manuscript addresses an interesting and medically important subject. The approach used to examine the effect of superantigen exposure on the response to IAV and/or IAV associated peptides is robust and should help gain a better understanding of the complex role superantigens and/or superinfections play in the response to influenza and possibly other viruses. Further, the findings of the importance of Vβ chain expression on the response to SEB exposure is novel.*”

We are grateful to the reviewer for his/her kind remarks.

**Part II – Major Issues: Key Experiments Required for Acceptance**

**Reviewer 1:** No response

**Reviewer 2:**

“*Rather oddly in the Discussion they immediately discuss results not mentioned in the Results section but shown only in the supplementary figures where rather different results were found using recombinant vaccinia for minigene priming. Then the normally immunodominant response to NP147 epitope was almost completely suppressed. This raises issues about use of T cell receptors and antigen presentation that ought to be explored experimentally in the Results section. They could either complete these experiments and put in the main section, strengthening the paper, or cut this additional data set out of this version and present those results later in a second paper, in which case they should put a caveat summarising these findings in this Discussion. The latter would be easier but the former would improve the paper.*”

We appreciate the reviewer’s point on the complexity of the experiment in which we used rVVs expressing flu minigenes. Therefore, as advised by the reviewer, we have removed this part and instead employed wild-type VacV in the interest of the comprehensiveness of our work. Looking at T_CD8_ responses to the three known peptide epitopes of VacV enabled us to explore the impact of SEB and to address T_CD8_ immunodominance in host responses to a very different virus. We will use the data on rVVs for a future submission on the priming pathways of IAV-specific T_CD8_ (please also refer to our responses to reviewer #1). Finally, we have moved the text describing the findings of VacV experiments to the end of the Discussion. We hope these modifications will be satisfactory to the reviewer.

**Reviewer 3:**

“*The authors should use a relevant model.*”

As indicated above, we have now enriched our manuscript, thanks to your input and that of the other reviewers, by including new results arising from additional vaccination and infection models. Please refer to new figures S2, 8 and 9 as well as text lines 421-429 among other revisions.

“*Time course experiments should be performed with SEB administration at various times (0, 3, 7, 10 days) post-infection (i.n.) or post vaccination (i.m.) to determine the effects of SEB on naïve and activated/memory CD8 T cells.*”

How exposure to SAgs impacts naïve and activated/memory T_CD8_ is definitely an important question. In our current manuscript, we examined recall responses to IAV using an established model of heterosubtypic immunity (Fig. 4 and Fig. 5). We found the outcome of SEB administration to depend on the time point at which this potent SAg was introduced. SEB injection 3 days before priming with PR8 raised the frequencies of CD127^high^KLRG1^low^ memory precursors and enhanced the anamnestic responses of SEB-reactive T_CD8_ (Fig. 4). By comparison, introducing SEB 3 days before boosting with SEQ12 diminished recall responses to IAV-derived epitopes (Fig. 5). This was accompanied by lower expression of Ki67 and higher Fas, LAG-3 and PD-1 levels consistent with a pro-apoptotic and/or exhausted phenotype. These findings prompted us to suggest that SAgs can have contrasting impacts on T_CD8_-mediated immunity to IAV depending on the naïve/activated/memory status of T_CD8_. Please also note that in a previous study, we reported that exposing preexisting IAV-specific memory T_CD8_ to SEB augments their cognate response [Meilleur CE, et al: *J Infect Dis* 2019, 219(8): 1307-17]. This was in the absence of a recall challenge. We have reiterated the above points in our revised manuscript.

We fully agree with the reviewer that kinetic studies will shed important light on how/when exposure to SAgs influences the responsiveness of naïve T_CD8_, recently activated (but previously naïve) T_CD8_ and recently re-activated memory T_CD8_. To address this question comprehensively, one would need to use several vaccination and infection models, which would require many mice, ample time and enough repeats for each experimental setting. This was really not possible for us given the duration of time we were granted for the revision of this manuscript. However, we did perform experiments in which the effect of SEB was tested when this SAg was either co-injected with PR8 to naïve mice or given 3 days after mice had been infected with PR8 and thus harbored recently activated anti-IAV T_CD8_. The results are depicted below. These experiments revealed that exposure to SEB three days after PR8 infection (and not at the time of PR8 inoculation) results in a decline in the NP_147_-specific response at its peak (*i.e.*, on day 10 post-PR8 infection), further suggesting that recently activated T_CD8_ behave differently than their naïve counterparts to SEB. The observed differences were either statistically significant or approached but did not reach statistical significance. In this setting, SEB and PR8 were co-administered i.n. We wish to repeat this experiment and also extend our study to several other timepoints and models, including vaccination with heat-inactivated PR8 and FluMist, as brilliantly suggested by the reviewer for a follow-up manuscript. We apologize for not including these relatively preliminary results in our current manuscript.

“*The majority of super infections or co-infections occur in the respiratory tract. It will be important to establish if administration of SEB at moderate levels in the respiratory tract affects IAV specific CD8 T cell responses.*”

Most definitely! As instructed by the reviewer, we conducted new experiments in which SEB and IAV were co-administered intranasally. To each mouse, we instilled 50 ng SEB in 25 µL of PBS, a dose we adopted from previously published articles [Herz U, et al: *Eur J Immunol* 1999, 29(3): 1021-31; Rajagopalan G, et al: *Infect Immun* 2006, 74(2): 1284-96]. Please see the new Fig. 8.

“*Supplementary Fig 2 & 3 are not mentioned in the results section but only in the discussion section”.*

The old Fig. S2 illustrated the results of vaccination with recombinant vaccinia viruses (rVVs) expressing flu minigenes. The results have now been removed, as instructed, and saved for another manuscript. The old Fig. S3, which is now our new Fig. S5, highlights changes in humoral anti-IAV responses, which was provided to reinforce a discussion point. Many journals no longer permit the discussion of any results that would be referred to as “data not shown”.

**Reviewer 4:**

“*The data, in its current form, is largely observational and does not provide solid evidence of a mechanism to support the observed effects. The authors provide reasonable explanations but no data to confirm.*”

In addition to several observations, we have provided some mechanistic insight into or at least clues for some of our findings. For instance, in addition to TCR Vβ profiling of cognate IAV-specific T_CD8_, we employed a mutant form of SEB (*i.e.*, SEB_N23A_), a fundamentally different SAg that is capable of binding to Vβ8.1-3 (*i.e.*, MAM), and a SAg that targets different TCR Vβ families in BALB/c mice *(i.e.*, SEA), we linked the responsiveness of select IAV-specific clones to their TCR Vβ makeup as opposed to their ranking in the immunodominance hierarchy. As another example, by performing some kinetic experiments and looking at proliferation and exhaustion markers, we provided some clues regarding how introduction of SEB at different time points during prime-boost immunization may affect the outcome. There is no question that some of our observations warrant further mechanistic investigations. Please also note that our revised manuscript also contains substantial new data in support of some of our observations. We hope the new additions will be satisfactory.

“*The work described in figure 4 has the potential, from a clinical standpoint, to be the most important observation. The effect of SEB exposure on the response in the lungs speaks to the clinical relevance of this work. In the current form, the authors suggest that the fact that they see a response in the spleen but not the lungs suggest that SEB exposure works locally and not at peripheral sites. While this is an important observation, no work was presented to describe whether local exposure in the lungs would result in alterations in the immune responses to IAV. This is a critical experiment and would significantly increase the strength of this work.*”

We completely agree with the reviewer and also thank him/her for his/her encouraging comments and valuable suggestion. As instructed, we performed new experiments in which SEB was administered intranasally (i.n.). We are pleased to report that as previously hypothesized, local administration of SEB augments the pulmonary immunodominant T_CD8_ response (please see new Fig. 8). We also believe that our additional experiments in which SEB-sufficient and -deficient *S. aureus* strains were used (new Fig. 9) should add more clinical relevance to this work. We hope the reviewer agrees. Finally, please note that our old Fig. 4 is now our new Fig. 7. It is simply renumbered due to the inclusion of additional results.

**Part III – Minor Issues: Editorial and Data Presentation Modification**

**Reviewer 1:** No response

**Reviewer 2:** None

**Reviewer 3:**

“*Does the amount of purified SEB administered in mice reflect the levels of SEB produced during bacterial infection?*”

This is truly an excellent question. We do not really know the answer since there are no firmly conclusive data available on local or systemic SEB levels during or after *S. aureus* infection in mice to our knowledge. We utilized SEB doses reported in many previously published papers.

“*Does SEB administration followed by SEQ12 result in increased TCD8 numbers?*”

Another great question! Yes, it does. To reach this conclusion, we performed new experiments in which primary responses to IAV epitopes were assessed after SEQ12 inoculation (please see new Fig. S3). This experiment enabled us to rule out the possibility that SEB injection shortly (3 days) before SEQ12 may unusually weaken the responsiveness of T_CD8_ to this particular strain irrespective of their naïve versus memory status. In this control experiment, naïve mice received SEB or PBS three days before they were inoculated with SEQ12. Similar to primary immunizations with PR8, NT60, HK, X31 and J-1 (Fig 1 and Fig 2), select cognate responses to SEQ12 (NP_147_ and HA_518_ in this case) were augmented. Therefore, the results shown in Fig 5B-5C reflect a unique feature of anamnestic T_CD8_ responses launched shortly after exposure to SEB.

“*[Old] P17 ‘N23 (Asn23) should also mediate the reactivity of SEB with mouse Vβ8.3’ should read ‘not mediate reactivity’?*”

As23 is important for the reactivity, and the SEB_N23A_ mutant should fail to react with Vβ8.3.

“*Fig 2C: As compared to SEB, mutant SEB N23A does not show significant decrease in NP147. The results section should be modified accordingly.*”

Thank you for bringing up this point. The main comparisons were between SEB- and PBS-treated cohorts and also between SEB_N23A_- and PBS-treated mice. However, as advised, we have now added a sentence to the text to clarify the comparison between SEB and SEB_N23A_. It reads “SEB was significantly more potent than SEB_N23A_ in inducing HA_518_- and HA_462_-specific responses, and a similar trend was observed in the case of NP_147_, which approached but did not reach statistical significance” (please refer to lines 464-466).

“*[Old] P16 ‘the above results demonstrate that systemic exposure to SEB expands, rather than deletes…’ As both the SEB and PR8 were injected i.p. this is just local exposure and not systemic exposure.*”

We have replaced the word “systemic” with the word “i.p.” in this sentence.

**Reviewer 4:**

“*The data presented in figure 1G demonstrating an increased response to NP147 loaded target cells is not convincing without inclusion of the control data. The authors should consider including the histograms from the naïve (PBS and SEB) mice.*”

Thank you for this excellent suggestion. We have now incorporated the results obtained in PBS- and SEB-treated control mice that received no IAV inoculation in the text (please refer to lines 401-410).
